# Diazotrophic *Paenibacillus beijingensis* BJ-18 Provides Nitrogen for Plant and Promotes Plant Growth, Nitrogen Uptake and Metabolism

**DOI:** 10.3389/fmicb.2019.01119

**Published:** 2019-05-29

**Authors:** Yongbin Li, Yunlong Li, Haowei Zhang, Minyang Wang, Sanfeng Chen

**Affiliations:** State Key Laboratory for Agrobiotechnology and College of Biological Sciences, China Agricultural University, Beijing, China

**Keywords:** diazotroph, ^15^N isotope enrichment, biological N_2_ fixation, colonization, GFP, wheat, maize, cucumber

## Abstract

Diazotrophic bacteria can reduce N_2_ into plant-available ammonium (NH_4_^+^), promoting plant growth and reducing nitrogen (N) fertilizer requirements. However, there are few systematic studies on the effects of diazotrophic bacteria on biological N_2_ fixation (BNF) contribution rate and host plant N uptake and metabolism. In this study, the interactions of the diazotrophic *Paenibacillus beijingensis* BJ-18 with wheat, maize, and cucumber were investigated when it was inoculated to these plant seedlings grown in both low N and high N soils, with un-inoculated plants as controls. This study showed that GFP-tagged *P. beijingensis* BJ-18 colonized inside and outside seedlings, forming rhizospheric and endophytic colonies in roots, stems, and leaves. The numbers of this bacterium in the inoculated plants depended on soil N levels. Under low N, inoculation significantly increased shoot dry weight (wheat 86.1%, maize 46.6%, and cucumber 103.6%) and root dry weight (wheat 46.0%, maize 47.5%, and cucumber 20.3%). The ^15^N-isotope-enrichment experiment indicated that plant seedlings derived 12.9–36.4% N from BNF. The transcript levels of *nifH* in the inoculated plants were 0.75–1.61 folds higher in low N soil than those in high N soil. Inoculation enhanced NH_4_^+^ and nitrate (NO_3_^-^) uptake from soil especially under low N. The total N in the inoculated plants were increased by 49.1–92.3% under low N and by 13–15.5% under high N. Inoculation enhanced activities of glutamine synthetase (GS) and nitrate reductase (NR) in plants, especially under low N. The expression levels of N uptake and N metabolism genes: *AMT* (ammonium transporter), *NRT* (nitrate transporter), *NiR* (nitrite reductase), *NR, GS* and *GOGAT* (glutamate synthase) in the inoculated plants grown under low N were up-regulated 1.5–91.9 folds, but they were not obviously changed under high N. Taken together, *P. beijingensis* BJ-18 was an effective, endophytic and diazotrophic bacterium. This bacterium contributed to plants with fixed N_2_, promoted plant growth and N uptake, and enhanced gene expression and enzyme activities involved in N uptake and assimilation in plants. However, these positive effects on plants were regulated by soil N status. This study might provide insight into the interactions of plants with beneficial associative and endophytic diazotrophic bacteria.

## Introduction

Biological nitrogen (N) fixation (BNF) is the major natural process through which atmospheric N_2_ is reduced to bioavailable NH_4_^+^, providing a large amount of natural N into cultivated agricultural systems (Galloway et al., 2008). In addition to symbiotic N_2_-fixing *Rhizobia* associated with legumes, the non-symbiotic diazotrophic bacteria are also important contributors to the N nutrition of non-legumes (Gupta et al., 2006). It is estimated that the microbial N accounts for roughly 30–50% of the total N in crop fields (Liu et al., 2017). The non-symbiotic diazotrophic bacteria are highly diverse and associated with plants in different ways. Some bacteria live in the rhizosphere and are designated rhizobacteria (Kloepper and Beauchamp, 1992). *Herbaspirilla seropedicae* strain Z67 mainly colonize on the riceroot surface and are usually called associative diazotrophic bacteria (Monteiro et al., 2012). *Paenibacillus polymyxa* WLY78 live inside the plant without causing damage and are classified as endophytic diazotrophic bacteria (Hao and Chen, 2017). Endophytic diazotrophic bacteria may have an advantage over associative diazotrophic bacteria and rhizobacteria, since they live within plant tissues where better niches are established for N_2_ fixation and assimilation of fixed N_2_ by the plant (Reinhold-Hurek and Hurek, 1998, 2011).

The well-known associative and endophytic diazotrophic bacteria include *Azospirillum* (Boddey et al., 1986), *Azoarcus* (Hurek et al., 2002), *Burkholderia* (Baldani et al., 2000), *Enterobacter* (Magnani et al., 2010), *Gluconacetobacter* (James et al., 2001), *Herbaspirillum* (Boddey et al., 1995). BNF quantification experiments show that associative and endophytic bacteria can fix N_2_ in plant tissues with higher efficiency (Carvalho et al., 2014). *G. diazotrophicus* inoculation enhanced sugarcane yield by providing 50–80% N from BNF (Boddey et al., 1995). It is estimated that an 18–28% of plant N derives from BNF of endophytic *Enterobacter* sp. strain (Mirza et al., 2001). Diazotrophic bacteria present in the mucilage of aerial roots contribute 29–82% of the N nutrition of Sierra Mixe maize (Van Deynze et al., 2018).

Although the positive effects of diazotrophic bacteria on plants are observed, little is known about plant response to inoculation with diazotrophic bacteria. Plant N metabolism is a complex process requiring some key enzymes. The plant genes *NR* (nitrate reductase), *NiR* (nitrite reductase), *GS* (glutamine synthetase) and *GOGAT* (glutamate synthase) play very important roles in N metabolism (Bloom et al., 1992; Lea and Miflin, 2003). The plant genes *AMT* (ammonium transporter) (Bloom et al., 1992) and *NRT* (nitrate transporter) (Sugiura et al., 2007) are involved in N uptake. It is shown that some endophytic fungi affect expression of N metabolism of plants (Yang et al., 2014).

*Paenibacillus beijingensis* BJ-18, isolated from wheat rhizosphere, was a N_2_-fixer (Wang et al., 2013). Inoculation with *P. beijingensis* BJ-18 promoted the growth of tomato seedlings (Xie et al., 2016) and increased wheat yield by 26.9% in field experiment (Shi et al., 2016), suggesting that this bacterium promotes plant growth. It was generally recognized that plant growth-promoting bacteria (PGPB) promoted plant growth by direct mechanisms (e.g., N fixation, phosphate solubilization, sequestering iron) and indirect mechanisms [e.g., indole-3-acetic acid (IAA), cytokinins, gibberellins] (Glick, 2012). However, the mechanisms utilized by *P. beijingensis* BJ-18 to promote plant growth were not clear. In this study, we investigated the colonization pattern and contributions of N_2_ fixation by *P. beijingensis* BJ-18 to plants, and the plant responses (N uptake and metabolism processes) to the infection.

## Materials and Methods

### Bacteria Strains and Growth Conditions

*Paenibacillus beijingensis* BJ-18 (accession number: JN873136), isolated from wheat rhizosphere, is a novel species with N_2_-fixing ability [1043 ± 12.9 nmol C_2_H_4_ (mg protein h)^-1^] (Wang et al., 2013). This bacterium has multiple antagonistic activities against plant pathogens and produces IAA (24.95 μg mL^-1^) (Xie et al., 2016). The bacterial suspension of *P. beijingensis* BJ-18 used in inoculation was prepared as follows. The *P. beijingensis* 1–18 cells were cultured overnight in Luria-Bertani (LB) broth at 30°C and 180 rpm, and then cells in the logarithmic growth phase were harvested by centrifugation and finally the pellet was suspended with sterile normal saline (0.89% w/v NaCl in double distilled water) to the final concentration at 10^8^ cells mL^-1^.

### Colonization of GFP-Tagged *P. beijingensis* BJ-18 in Wheat, Maize, and Cucumber Tissues

The recombinant plasmid pGFP300 carrying *gfp* gene (Hao and Chen, 2017) was introduced into *P. beijingensis* BJ-18 by electrotransformation (Zhang et al., 2013), yielding GFP-tagged *P. beijingensis* BJ-18. And the physiological ability of GFP-tagged *P. beijingensis* BJ-18 was not changed, compared with wild-type *P. beijingensis* BJ-18 (data not published). The GFP-tagged *P. beijingensis* BJ-18 suspension was obtained as described above. Plump seeds of wheat “Jimai 22” (Shandong Runfeng Seed Industry Co., Ltd), maize “Zhengdan 958” (Henan Shangke Seed Co., Ltd.) and cucumber “Zhongnong 8” (Beijing Shengfeng Garden Agricultural Technology Co., Ltd) were surface-disinfected with 10% sodium hypochlorite for 10 min, followed by rinsing with sterilized water three times, and grown on the sterile petri dishes containing moist filter papers in darkness at room temperature (25°C) for 3–5 days, respectively. These plant seedlings had two treatments: inoculation with GFP-tagged *P. beijingensis* BJ-18 (E+) and mock inoculation (E-). For inoculation, the plant seedlings were soaked in bacterial suspension (10^8^ cells mL^-1^) of GFP-tagged *P. beijingensis* BJ-18 for 30 min to facilitate colonization. For mock inoculation, the plant seedlings were soaked in sterilized deionized water for 30 min. The germinated wheat, maize and cucumber seeds were, respectively, sown in sterile flask (3 seeds per flask, 6 cm in diameter and 10 cm in height) containing 100 mL 1/2 × Murashige and Skoog semisolid agar medium (Prod No: M519, PhytoTechnology Laboratories, Shawnee Mission, United States) (Murashige and Skoog, 1962). Then these seedlings were grown in the light growth chamber (27°C, 70% humidity and 16 h day/8 h night, with light at 250 μmol m^-2^ s^-1^). The GFP-tagged *P. beijingensis* BJ-18 in plant tissues was observed at 2 weeks after inoculation by confocal laser scanning microscopy (CLSM, Olympus FluoView^TM^ FV1000 confocal microscope, Olympus Corporation, Tokyo, Japan). These images were collected using FV10-ASW software (03.01.02.02, Olympus Europa Holding GmbH, Hamburg Germany) and processed in Adobe Photoshop CC 2015 and Adobe Illustrator CS6 (Adobe, San Jose, CA, United States).

### Plant Culture and Collection

Seedling growth assays were performed in plastic pots (diameter of 35 cm; height of 25 cm) filled with 5 kg non-sterile soil which was top soil (0–20 cm depth) taken from the Shangzhuang Experimental Station of China Agricultural University, Beijing, China (40°08′12.15″ N, 116°10′44.83″ E, 50.21 m above sea level). The soil was low N-content sandy loam (N_min_, 7.8 mg kg^-1^; Olsen-P, 7.3 mg kg^-1^; NH_4_OAc-K, 115.8 mg kg^-1^; O.M., 7.2 g kg^-1^; pH 7.7; E.C., 0.4 dS m^-1^). The soil was air-dried, crushed, and screened by a 2 mm sieve to remove debris and reduce heterogeneity for cultivating plants: wheat, maize and cucumber. Before planting, P (Na_2_HPO_4_) and K (KCl) fertilizer were applied to soil as base fertilizers at amounts of 50 mg P and 17 mg K per kg soil, respectively, based on the recommendation by Ke et al. (2018). The microelements were not applied to soil during plant growth.

The experimental design was randomized with factorial arrangement (a 2 × 2 factorial design) in three replications with bacterial factor at two levels and N factor at two levels. Three different plants (wheat, maize, and cucumber) were chosen to obtain an objective conclusion. Therefore, the experiments had twelve treatments.

The seeds (wheat, maize, and cucumber) were germinated as described above. After germination, vigorous and homogenous seedlings were chosen for transplanting into plastic pots. These plant seedlings had two treatments: inoculation with *P. beijingensis* BJ-18 (E+) and mock inoculation (E-). For inoculation, the plant seedlings were soaked in bacterial suspension (10^8^ cells mL^-1^) of *P. beijingensis* BJ-18 for 30 min to facilitate colonization. For mock inoculation, the plant seedlings were soaked in sterilized deionized water. Then the inoculated seedlings and un-inoculated seedlings were, respectively, transplanted into pots (cucumber: 4 seedlings per pot; maize: 2 seedlings per pot; wheat: 4 hills per pot and 10 seedlings per hill). On day 7, 80 ml of the bacterial suspension was applied to pot containing inoculated seedlings and 80 mL of sterile water was applied to pot containing non-inoculated seedlings. Each of inoculation and mock inoculation treatments had three replicates.

There were two levels of N treatments: high N level (250 mg N kg^-1^ soil) and low N level (83 mg N kg^-1^ soil). The ^15^N-labeled (NH_4_)_2_SO_4_ (10.16% ^15^N atom, Shanghai Research Institute of Chemical Industry, China) was applied to soils in all pots. The N fertilizer was added in three separate applications: the first application was done before planting as base fertilizer (approximately 33.3% of total N), and successive two applications (approximately 33.3% of total N per time) were made on day 7 and 14 after transplanting, respectively. Pots were placed in the greenhouse under optimum conditions (15 h light/9 h dark cycle, 25–30/15–20°C day/night temperature and 40% day/60% humidity). The seedlings were regularly watered (tap water) to 40% relative soil moisture by weighing method every 5 days.

The samples of wheat, maize, and cucumber were harvested from each treatment on day 35 after transplanting, respectively.

Firstly, the whole seedling was uprooted, and then shoot and root samples were separated and washed with deionized water to remove the adhering soil. Shoot and root samples were oven-died at 105°C for 30 min to inactivate the enzyme, respectively, followed by 65°C until constant weight for dry weight analysis. Then, the oven-dried samples were grinded, screened by a 1 mm sieve, and stored in zip-lock bag for plant N content and ^15^N enrichment determination. Afterward, the remaining samples were immediately frozen in liquid N and then maintained at -80°C until further analysis.

### Bacterial Cell Concentration Within Plant Tissues

The cell densities of diazotrophic *P. beijingensis* BJ-18 within the inoculated plant tissues were estimated by qPCR according to the method described by Rasmussen et al. (2007). Primers for qPCR of the *nifB* from *P. beijingensis* BJ-18 included *nifB* F (5′-GAAGGTGAGAGTGAGGATGG-3′) and *nifB* R (5′-TTGCTTCAGGCTCATCTCC-3′). qPCR was performed with plant genomic DNA as template which was extracted form plant seedlings using DNA Kit [TianGen Biotech (Beijing) Co., Ltd.]. The 129 bp PCR product was ligated to the PMD 19-T vector (Takara, Otsu, Japan) and then introduced into *Escherichia coli* JM109. The introduced *E. coli* JM109 was grown in liquid LB medium, and the recombinant plasmids carrying *nifB* fragment were extracted and purified using TIANprep Mini Plasmid Kit [TianGen Biotech (Beijing) Co., Ltd.]. A standard curve was generated for each run 10-fold dilution series from 2 × 10^1^ to 2 × 10^7^ copies. The plant genomic DNA isolated from each of the different treatments was mixed with SYBR^®^ Premix Ex TaqTM (Takara, Kyoto, Japan), primer pairs and ddH_2_O in a total volume of 20 uL for qPCR on a 7500 Real-Time PCR System (Applied Biosystems, Foster City, CA, United States). Ct values were measured to quantify initial amounts of target DNA.

### Quantification of Biological N_2_ Fixation Contribution

In this study, BNF contribution of *P. beijingensis* BJ-18 to plants was quantified by the method of ^15^N isotope dilution technique. N content and ^15^N enrichment in plant tissues were determined by DELTA V Advantage isotope ratio mass spectrometer (Thermo Fisher Scientific, Inc., United States). The plants without *P. beijingensis* BJ-18 inoculation were used as references to calculate the BNF contribution. The BNF contribution was calculated according to formula 1 described by Boddey and Knowles (1987):

(1) $$\%\mathrm{Ndfa}=\left(1-\frac{{\%}^{15}\mathrm{Na}.e._{I}}{{\%}^{15}\mathrm{Na}.e._{\mathrm{UI}}}\right)\times 100$$

Where, %Ndfa is the percentage of N derived from air and percent ^15^Na.e. (%^15^N atom excess) is the enrichment of the inoculated (I) and un-inoculated (UI) plants, respectively.

### Determination of the Concentration of Free NH_4_^+^, NO_3_^-^, and Activities of GS and NR

In order to determine the concentrations of the free ammonium (NH_4_^+^) and nitrate (NO_3_^-^), fresh plant tissues were ground with a mortar on ice in extraction buffer. The buffer consisted of 10 mM imidazole, 50 mM Tris-HCl (pH 8.0), and 0.5% (w/v) *β*-mercaptoethanol. After grinding, the samples were centrifuged at 12,000 × *g* for 20 min at 4°C and the supernatant was collected for free NH_4_^+^ and NO_3_^-^ determination (Oliveira et al., 2002; Yang et al., 2014). Free NH_4_^+^ concentration [μg g^-1^ fresh weight (FW)] in the supernatant was assayed using the Berthelot color reaction method (Gordon et al., 1978), and free NO_3_^-^ concentration (μg g^-1^ FW) in the supernatant was determined using the Griess method (Eckhardt et al., 1999).

For analysis of GS activity, fresh plant tissues were ground with a mortar in pre-cold extraction buffer (containing10 mM MgSO_4_, 2 mM dithiothreitol, 70 mM 3 (n-morpholino) propane-sulfonic acid (pH 6.8), 5 mM glutamate, 10% (v/v) ethanediol, 0.1% (v/v) TritonX-100). The extracts were centrifuged at 12,000 × *g* for 30 min at 4°C and the supernatant was collected for plant GS activity determination using NH_2_OH as a substrate, and the amount of γ-glutamyl hydroxamate (GHA, ug^-1^ FW min^-1^) released was determined spectrophotometrically at 540 nm according to the method of Yang et al. (2014).

To measure NR activity, fresh plant tissues were ground on ice in extraction buffer consisting of 25 mM phosphate buffer (pH 7.5, a mixture of K_2_HPO_4_ and KH_2_PO_4_), 5 mM cysteine and 5 mM EDTA-Na_2_. The mixture was centrifuged at 4,000 × *g* and 4°C for 10 min and the supernatant was collected. NR activity was measured spectrophotometrically at 540 nm according to Yu and Zhang (2012). NR activity was expressed as μg NO_2_^-^ g^-1^ FW h^-1^.

### Quantitative Real-Time PCR Analysis of Plant Genes and *nifH* in Plant Roots and Shoots

Total RNA was extracted from plant tissues using RNAiso Plus reagent (RaKaRa, Kyoto, Japan). Then, RNA was digested with DNase I and reversely transcribed into cDNA using PrimeScript^TM^ RT reagent kit (RaKaRa, Kyoto, Japan). Gene expression levels were determined by quantitative real-time PCR (qRT-PCR) analysis. The specific primers for qRT-PCR were shown in Table 1. The plant housekeeping gene *actin* was used as plant internal control, and the bacterial 16S rRNA was used as bacterium internal control. The relative expression of the target genes were calculated according to the standard comparative C(t) method (Livak and Schmittgen, 2001). Each treatment had three biological replicates, with three technical replicates for each biological replicate.

**Table 1:** Primers sequence and accession number in NCBI.

Primer	Primer sequence 5′-3′	Size (bp)	NCBI Accession No.	References
*CsAMT1*	TTCTCTATCAGTGGGCTTTCG	141	AY642427	This study
	AGAACCAATGGGACACAACC			
*CsAMT3*	AAGGTAGACGACACAATGG	109	XM_004138819	This study
	CGTAGAAGATGTTGTTGAGG			
*CsNRT 1.3*	CACAAGCCTTCAGAGAATTGG	131	JX206800	This study
	TCAACCAGAAAGCACTTATACG			
*CsNR1*	GCACAACTCAGACCAATCC	103	HM755943	This study
	GATGAGAATGCTGTCCATACC			
*CsNR2*	TGTGCGTGTATTCAGATTCG	132	HM755944	This study
	GTGCTAGAGGGCGTATAGG			
*CsNiR*	AGGATTGGTAGCTTGCACTGG	105	EF397679	This study
	ACTGTGACTCGCCGTTGC			
*CsGS1*	ATGAGGGAAGAAGGAGGTTACG	147	JQ277263	This study
	AGAGAAGGTGTGGATGTCAGC			
*CsGOGAT*	GGCTGCTCAAGGAAAGGAACC	127	DQ641082	This study
	TGCTGGATTTGTCACCTGTGC			
*TaAMT1.1*	ACAGCTTCTTCCTCTTCC	105	AY525637	This study
	CCGAGTAGATGAGGTAGG			
*TaNRT1.1*	ATGCCAGGTTGTCATTGC	135	AY587265	This study
	CCGAGTCCAGTTGTATGC			
*TaNRT2.1*	TGGACTCCGAGCACAAGG	104	AF288688	This study
	GACGAAGCAGGTGAAGAAGG			
*TaNRT2.3*	TGGTCAGAGGAGGAGAAGG	101	AY053452	This study
	GTGGCGAGGATAACATTGC			
*TaNR*	ATACACCATGAAAGGATACG	126	KY244026	This study
	TACTTGTTCGGCTTCTCC			
*TaNiR*	CTACACCAACCTCCTCTCC	138	FJ527909	This study
	GCCAGGTCGTTGATATGC			
*TaGS1*	CCTTGTCATGTGCGATTGC	135	DQ124211	This study
	GTGTACTCCTGCTCGATACC			
*Ta GOGAT*	AAACCAAGGGACCTCAGTATTC	150	DW986179	This study
	AATGACCACCACCTTCTTACC			
*ZmAMT1;1a*	CATCGTCGGAAGGTGTGG	109	GRMZM2G175140	This study
	TTGGATGATGAGCAGTGACC			
*ZmAMT1;1b*	CTACGACTTCTTCCTATACC	111	GRMZM2G118950	This study
	CGGAGTAGATGAGGTAGG			
*ZmNrt2.1*	TGGACTCAGAGCACAAGG	102	AY129953	This study
	CGAAGCAGGTGAAGAAGG			
*ZmNR*	GCCAGCATTGAAGGGAAG	106	M77792	This study
	GCTCGTTCTTGAAGTAGACC			
*ZmGS1-3*	CTTGTGATGTGCGATTGC	129	X65928	This study
	CTCCTGCTCAATACCATACC			
*ZmGS1-4*	AGGCATCAACATCAGTGG	117	X65929	This study
	AGAATGTAGCGAGCAACC			
*Zm GOGAT*	*CGCTCTTCTGGCAACTGG*	133	M59190	This study
	*CACCTTCCTGTAGTCTGATTCG*			
*CsACTIN*	AGAGATGGCTGGAATAGAAC	333	DQ641117	Wei et al. (2015)
	CTGGTGATGGTGTGAGTC			
*ZmACTIN*	CATGGAGAACTGGCATCACACCTT	118	J01238.1	Galli et al. (2013)
	CTGCGTCATTTTCTCTCTGTTGGC			
*TaACTIN*	GTCGGTGAAGGGGACTTACA	187	AB181991.1	Moloudi et al. (2013)
	TTCATACAGCAGGCAAGCAC			
*nifB*	GAAGGTGAGAGTGAGGATGG	88	MH202771	This study
	TTGCTTCAGGCTCATCTCC			
*nifH*	GCAACAGTCGGAATACGG	136	MH555146	This study
	TTGGGTCACGGTCATACG			


### Statistical Analysis

Statistical tests were performed using SPSS software version 20 (SPSS Inc., Chicago, IL, United States). Two-way analysis of variance (ANOVA) was employed to check the significant differences between treatments. Means of different treatments were compared using the least significant difference (LSD) at 0.05 or 0.01 level of probability. Graphs were prepared using SigmaPlot software version 12.5 (Systat Software, Inc., CA, United States).

## Results

### Colonization of GFP-Tagged *P. beijingensis* BJ-18 in Wheat, Maize, and Cucumber Tissues

**FIGURE 1:**
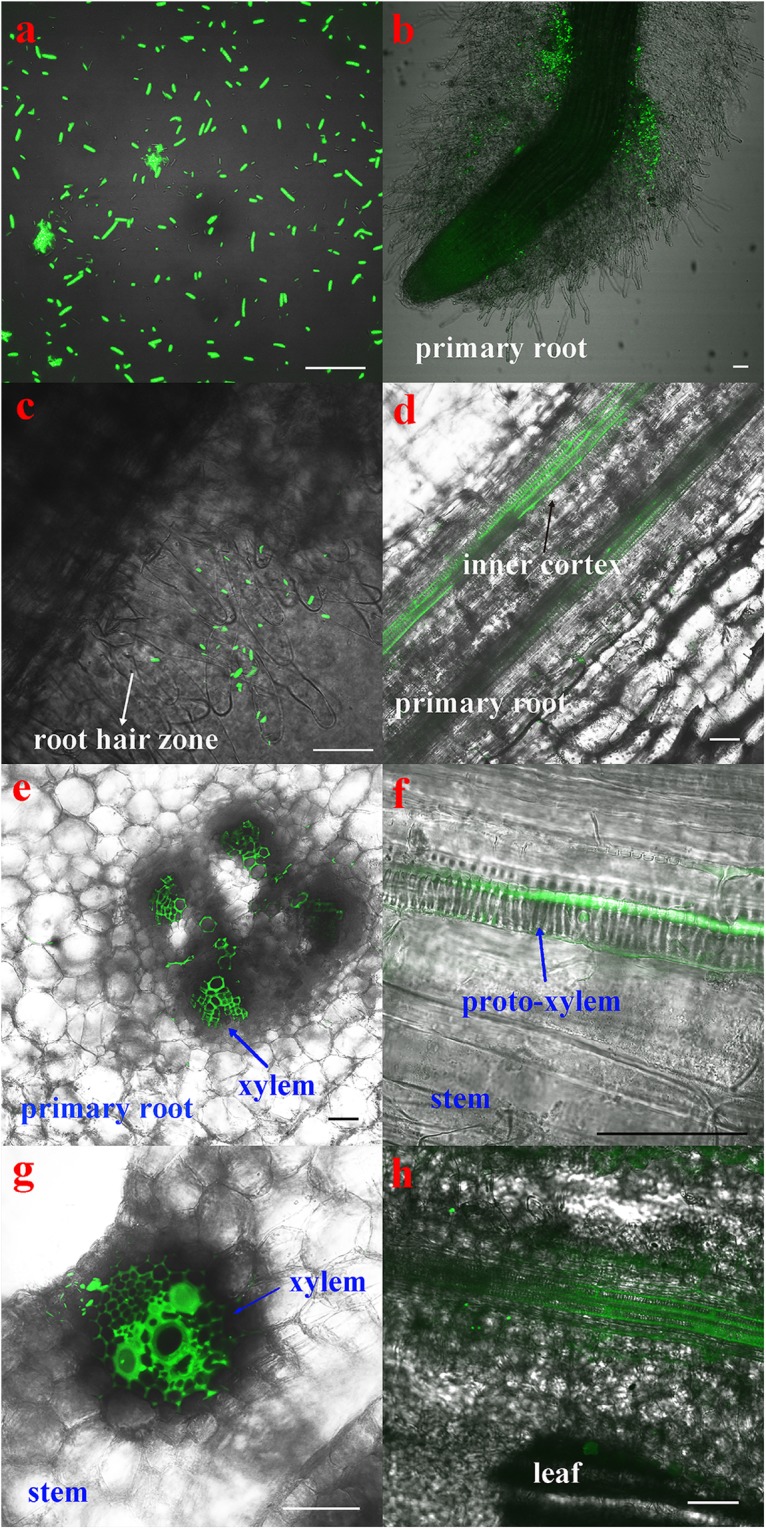
Colonization of the GFP-tagged *P. beijingensis* BJ-18 in the seedlings of wheat. Wheat seedlings were grown in the presence of GFP-tagged *P. beijingensis* BJ-18 for 2 weeks. Images were taken with a fluorescent microscope. Excitation was at 488 nm. **(a)** Confocal image of the GFP-tagged *P. beijingensis* BJ-18 cells; **(b–e)** Colonization patterns in the root; **(f,g)** Colonization patterns in the stem; **(h)** Colonization patterns in the leaf. Bars represent 50 μm.

**FIGURE 2:**
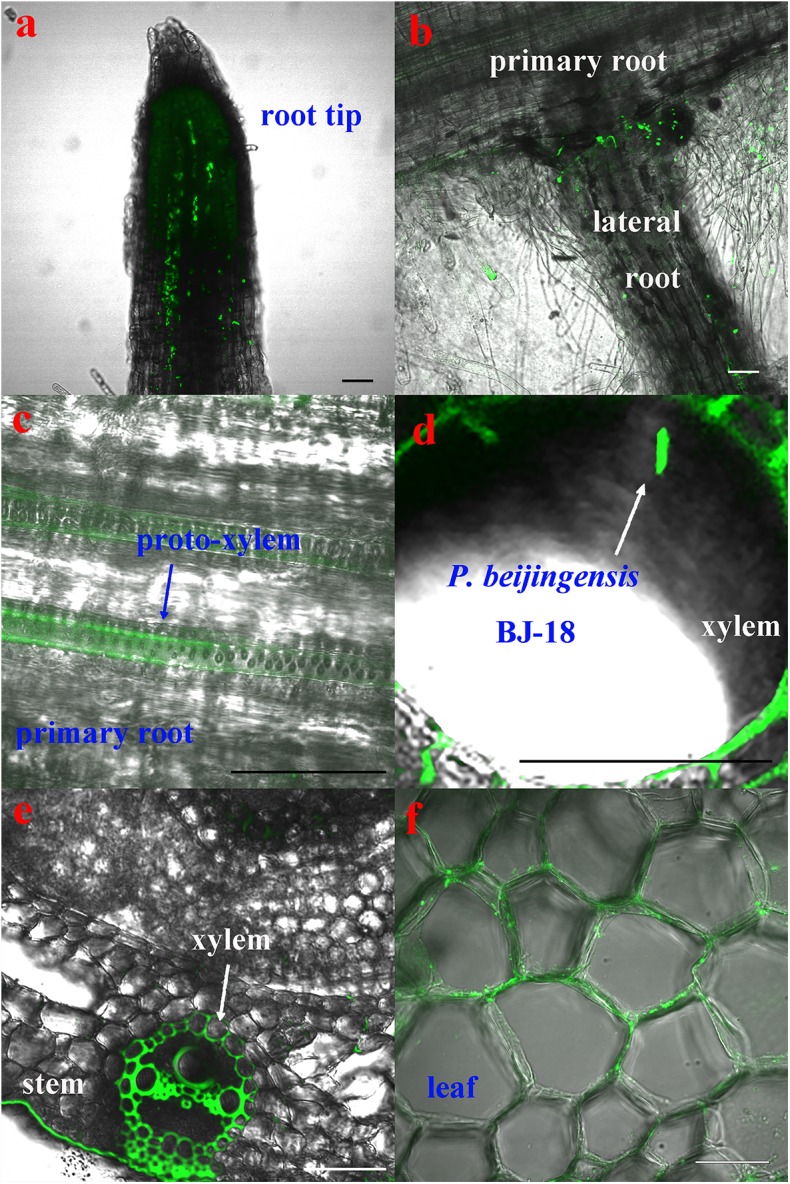
Colonization of the GFP-tagged *P. beijingensis* BJ-18 in the seedlings of maize. Maize seedlings were grown in the presence of GFP-tagged *P. beijingensis* BJ-18 for 2 weeks. Images were taken with a fluorescent microscope. **(a–c)** Colonization patterns in the root; **(d,e)** Colonization patterns in the stem; **(f)** Colonization patterns in the leaf. Bars represent 50 μm.

**FIGURE 3:**
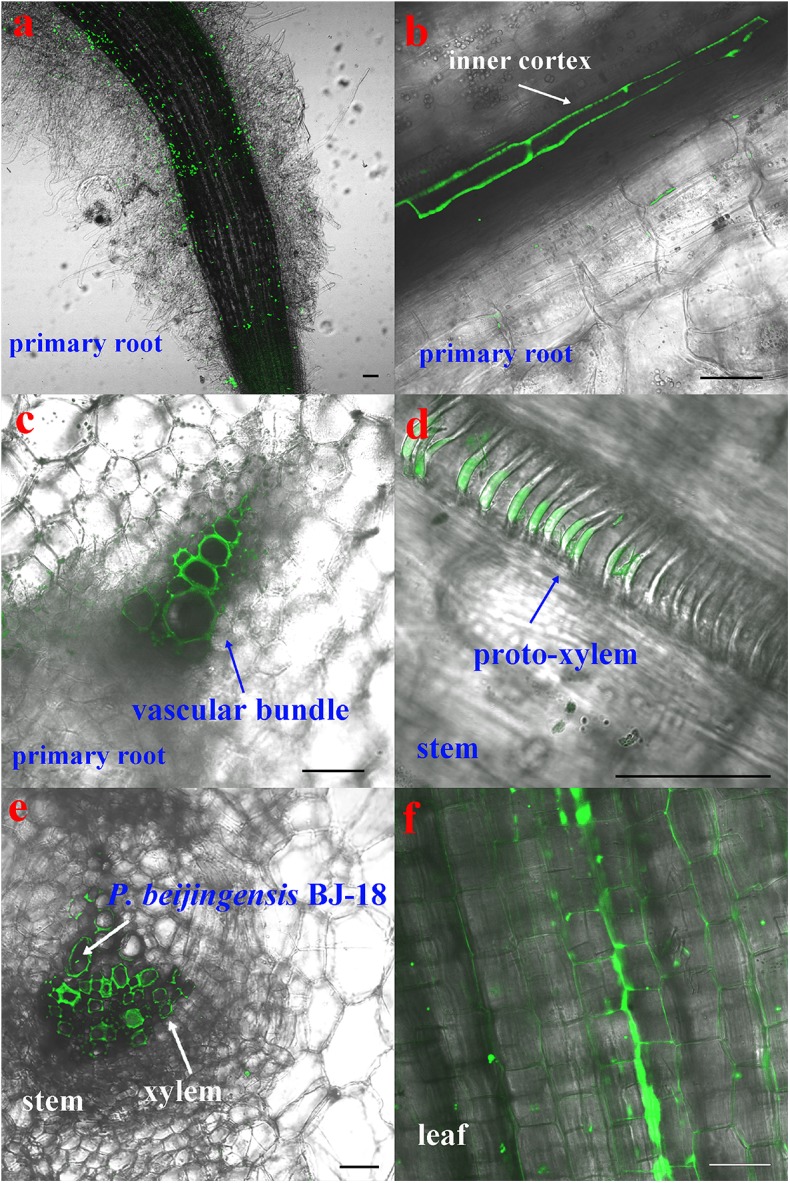
Colonization of the GFP-tagged *P. beijingensis* BJ-18 in the seedlings of cucumber. Cucumber seedlings were grown in the presence of GFP-tagged *P. beijingensis* BJ-18 for 2 weeks. Images were taken with a fluorescent microscope. **(a–d)** Colonization patterns in the root; **(e)** Colonization patterns in the stem; **(f)** Colonization patterns in the leaf. Bars represent 50 μm.

The CLSM observation showed that the GFP-tagged *P. beijingensis* BJ-18 cells emitted bright green fluorescence (Figure 1a). The GFP-tagged *P. beijingensis* cells were found to colonize on the surface of the primary roots and the root hair zone of wheat (Figure 1b,c). The bacterial cells were found to be distributed within cortex of wheat primary roots (Figure 1d) and colonized in the wheat vascular bundle (Figure 1e). Moreover, bacterial cells were observed in the vascular bundle of wheat stem (Figure 1f,g) and in wheat leaf vein (Figure 1h). In the maize seedlings, the *P. beijingensis* cells were found to colonize the surface of the primary roots (Figure 2a), and on the junction of the primary and lateral roots (Figure 2b). The bacteria cells were found within the intercellular spaces and xylem vessels of maize roots and stems (Figure 2c–e) and leaves (Figure 2f). The colonization pattern in cucumber was similar to those obtained in wheat and maize seedlings. The bacterial cells were found to colonize the surface of the primary roots and the root hair zone (Figure 3a). The *P. beijingensis* cells were in cortex (Figure 3b) and vascular bundle (Figure 3c) of cucumber roots. As shown in Figure 3d,e, bacterial cells invaded the xylem vessels of cucumber stem. Moreover, the bacterial cells were found within the cucumber leaves (Figure 3f).

Taken together, *P. beijingensis* cells colonized on the surface of roots and within roots, stems and leaves of wheat, maize, and cucumber, indicating that the colonization patterns in the three plants were similar.

### Concentration of Diazotrophic *P. beijingensis* BJ-18 in Wheat, Maize, and Cucumber Tissues

**FIGURE 4:**
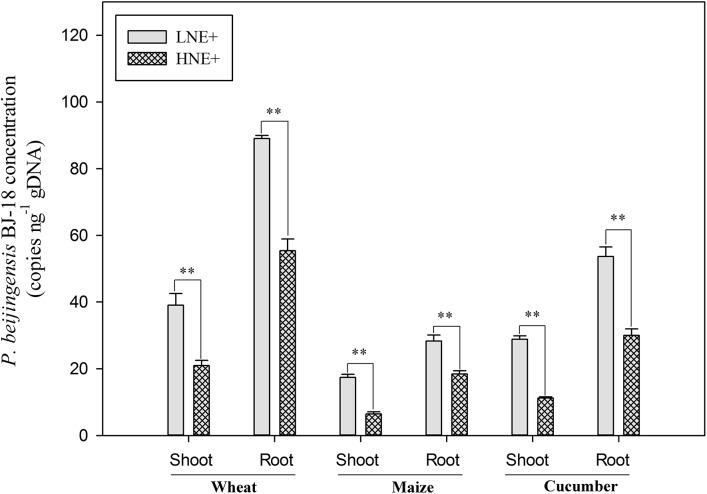
The cell concentrations of *P. beijingensis* BJ-18 in the inoculated shoots and roots of wheat, maize, and cucumber seedlings in high N (HN) and low N (LN) levels. The concentrations of *P. beijingensis* BJ-18 are represented by *nifB* gene copies ng^-1^ total genomic DNA. Values are given as mean of three independent biological replicates, and single asterisks or double asterisks (^∗^ or ^∗∗^) indicate significant differences between HNE+ and LNE+ treatments determined by LSD at *P* < 0.05 or *P* < 0.01. The bars represent the standard error. LNE+ indicates plants grown in low N level of soils and inoculated with *P. beijingensis* BJ-18; HNE+ indicates plants grown in high N level of soils and inoculated with *P. beijingensis* BJ-18.

The concentration of *P. beijingensis* BJ-18 in the inoculated plants at 35 day after inoculation was assessed using qPCR and was expressed as the number of copies of the specific *nifB* genes per total (plant seedlings + bacteria) genomic DNA. As shown in Figure 4, copy numbers of *nifB* gene in the inoculated plant tissues under low N level were much higher than under high N level. Wheat had the highest copy numbers of *P. beijingensis nifB* gene, followed by cucumber and then maize under both low N and high N levels. Copy numbers of *nifB* gene in roots of the three plants were much higher (62.5–185.3%) than in shoots under both low N and high N levels. These data suggested that the concentrations of the bacterial cells in plant tissues were related to soil N levels, plant species and plant tissues.

### Plant Growth Promotion

**FIGURE 5:**
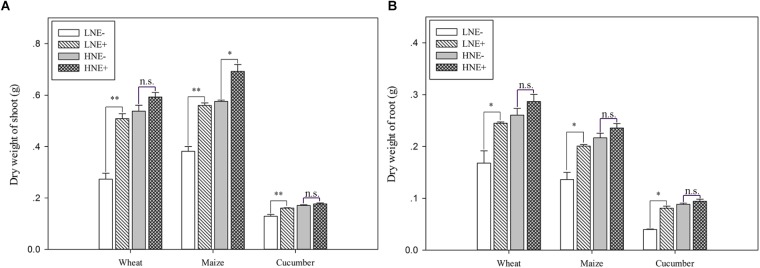
Dry weight (DW) in shoots **(A)** and roots **(B)** of wheat, maize, and cucumber seedlings inoculated with *P. beijingensis* BJ-18 under high N (HN) and low N (LN) levels. Values are given as mean of three independent biological replicates, and single asterisks or double asterisks (^∗^ or ^∗∗^) indicate significant differences between inoculated (E+) and un-inoculated (E-) plants determined by LSD at *P* < 0.05 or *P* < 0.01. The bars represent the standard error. The wheat dry weight represents five seedlings per pot, and the dry weight of maize and cucumber represents one seedling per pot. LNE- indicates plants grown in low N level of soil and un-inoculated with *P. beijingensis* BJ-18; LNE+ indicates plants grown in low N level of soils and inoculated with *P. beijingensis* BJ-18; HNE- indicates plants grown in high N level of soil and un-inoculated with *P. beijingensis* BJ-18; HNE+ indicates plants grown in high N level of soils and inoculated with *P. beijingensis* BJ-18.

To assess the impacts of *P. beijingensis* inoculation on plant growth, the biomass (dry weight) was analyzed in inoculated and un-inoculated plants under low N and high N levels. Dry weights of wheat shoots and in roots under low N were increased by 86.1 and 46.0%, respectively. Dry weights of maize shoots and in roots under low N were increased by 46.6 and 47.5%, respectively. Dry weights of cucumber shoots and in roots under low N were increased by 103.6 and 20.3%, respectively. The data suggested that *P. beijingensis* BJ-18 could efficiently promote the growth of the three plants (Figure 5). The increased shoot and root biomass by *P. beijingensis* BJ-18 inoculation under low N condition were significantly higher than those under high N condition, consistent with the concentration of *P. beijingensis* BJ-18 in these plant tissues under different N conditions.

### Quantification of BNF in the *P. beijingensis*-Inoculated Wheat, Maize, and Cucumber

To estimate the contribution of BNF, ^15^N isotope enrichment analysis was conducted to analyze inoculated seedling of wheat, maize and cucumber grown in soil contain ^15^N-labeled (NH_4_)_2_SO_4_ as N fertilizer in greenhouse conditions, in comparison with un-inoculated plants. As shown in Table 2, the roots and shoots of seedlings inoculated with *P. beijingensis* BJ-18 had significantly lower δ^15^N value than un-inoculated seedlings under both low and high N conditions, suggesting that these plants derived a portion of N from atmospheric N_2_. The plant N derived from the atmosphere (%Ndfa) ranged from 18 to 36.4% under low N level and from 12.9 to 30.5% under high N level. Among the three plants, wheat showed maximum %Ndfa (30.5 and 36.4%) under both high N and low N levels, followed by cucumber (25.4 and 27.8%) and then maize (12.9 and 20.9%). These data suggest that the contribution of BNF was closely related to soil N levels and to plant species. These results were in agreement with the concentration of *P. beijingensis* BJ-18 within plants and the plant biomass under different N levels.

**Table 2:** ^15^N isotope enrichment determination of biological N_2_ fixation rate in inoculated plants grown in soils containing high N and low N.

Treatments			δ15N value (versus at-air)		%Ndfa	
		
			High N	Low N	High N	Low N
	Shoot	E-		10952 ± 1251a	3680 ± 261a
Cucumber		E+	7641 ± 727a	2656 ± 176b	25.4 ± 0.4b	27.8 ± 0.4a
	Root	E-	12926 ± 443a	4282 ± 738a	—	—
		E+	9457 ± 197b	2935 ± 503b	26.8 ± 1.1b	31.4 ± 0.9a
	Shoot	E-	6662 ± 1076a	3056 ± 299a	—	—
Wheat		E+	4596 ± 631a	1940 ± 171b	30.5 ± 1.8b	36.4 ± 0.6a
	Root	E-	6123 ± 316a	3573 ± 234a	—	—
		E+	4758 ± 274b	2614 ± 172b	22.3 ± 0.9b	26.9 ± 0.4a
	Shoot	E-	5383 ± 434a	2222 ± 137a	—	—
Maize		E+	4677 ± 296a	1756 ± 88b	12.9 ± 1.5b	20.9 ± 1.0a
	Root	E-	4523 ± 174a	2357 ± 292a	—	—
		E+	3885 ± 160a	1932 ± 235b	14.1 ± 0.4b	18.0 ± 0.3a


*Values are given as mean ± SE of three independent biological replicates. Different letters indicates indicate significantly differences in δ15N value inoculated (E+) and un-inoculated (E-) plants according to the LSD test (*P* < 0.05); Different letters indicate significantly differences in %Ndfa between high N and low N according to the LSD test (*P* < 0.05); δ^*15*^N value: percent atom excess ^*15*^N; %Ndfa: percent N derived from atmosphere.*

### Transcript Levels of *nifH* Gene

**FIGURE 6:**
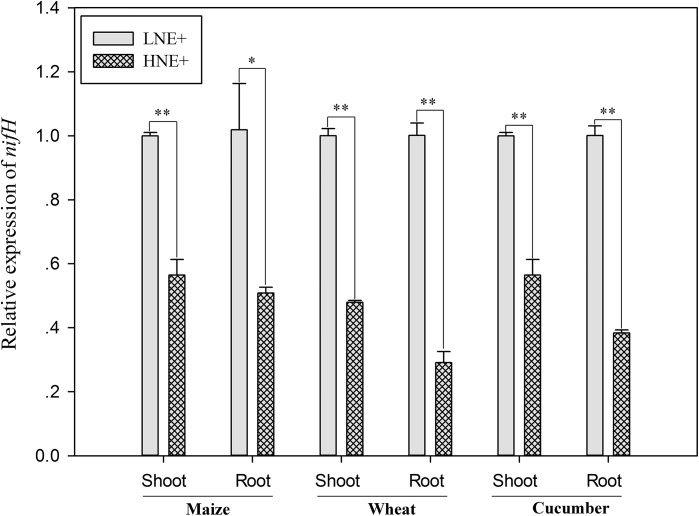
The *nifH* transcript levels of *P. beijingensis* BJ-18 in shoots and roots of wheat, maize and cucumber seedlings inoculated with *P. beijingensis* BJ-18 under high N (HN) and low N (LN) levels. Values are given as mean of three independent biological replicates, and single asterisks or double asterisks (^∗^ or ^∗∗^) indicate significant differences between HNE+ and LNE+ treatments determined by LSD at *P* < 0.05 or *P* < 0.01. The bars represent the standard error. LNE+ indicates plants grown in low N level of soils and inoculated with *P. beijingensis* BJ-18; HNE+ indicates plants grown in high N level of soils and inoculated with *P. beijingensis* BJ-18.

To investigate whether *nif* genes coding nitrogenase of *P. beijingensis* BJ-18 were expressed within plant tissues, the transcript levels of *nifH* gene, one of *nif* genes coding Fe protein of nitrogenase, were quantified. As shown in Figure 6, transcripts of *nifH* under low N were up-regulated in wheat shoots and roots by 1.09 and 1.61 folds, respectively, in maize shoots and roots by 0.77 and 1.0 folds, respectively, and in cucumber shoots and roots by 0.75 and 1.61 folds, respectively, compared to those under high N. The results suggested that soil N status affected transcript level of *P. beijingensis nifH*. The data were consistent with BNF rate and the concentration of *P. beijingensis* BJ-18 in plants grown in soils containing different N levels.

### Promotion of N Uptake and Total N Content in Plants by Inoculation With *P. beijingensis* BJ-18

**FIGURE 7:**
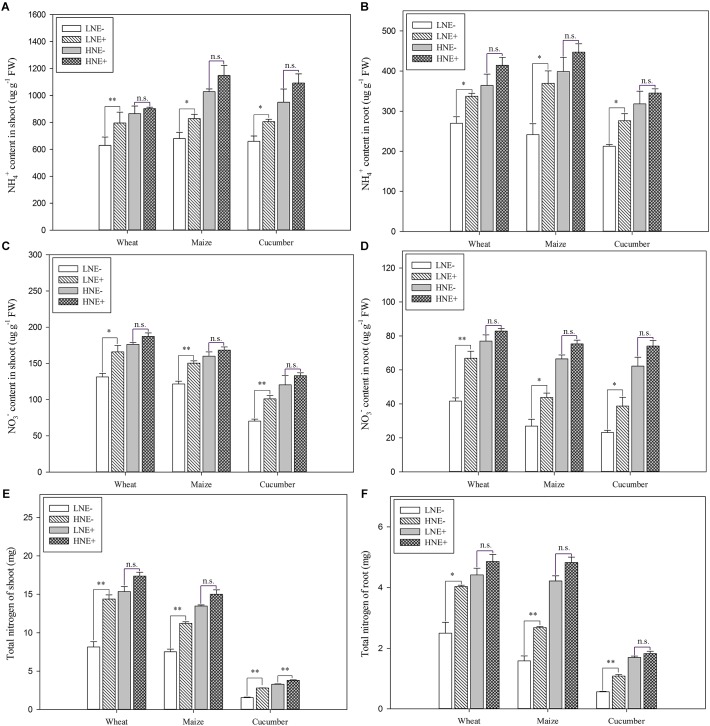
Ammonium (NH_4_^+^) (**A**: shoot; **B**: root), nitrate (NO_3_^-^) (**C**: shoot; **D**: root) and total N (**E**: shoot; **F**: root) content in shoots and roots of wheat, maize and cucumber seedlings inoculated with *P. beijingensis* BJ-18 under high N (HN) and low N (LN) levels. Values are given as mean of three independent biological replicates, and single asterisks or double asterisks (^∗^ or ^∗∗^) indicate significant differences between inoculated (E+) and un-inoculated (E-) plants determined by LSD at *P* < 0.05 or *P* < 0.01. The bars represent the standard error. LNE- indicates plants grown in low N level of soil and un-inoculated with *P. beijingensis* BJ-18; LNE+ indicates plants grown in low N level of soils and inoculated with *P. beijingensis* BJ-18; HNE- indicates plants grown in high N level of soil and un-inoculated with *P. beijingensis* BJ-18; HNE+ indicates plants grown in high N level of soils and inoculated with *P. beijingensis* BJ-18.

Compared to un-inoculated shoots and roots under low N level, a significant increase of free NH_4_^+^ concentration was observed in shoots of wheat (26.6%), maize (21.9%) and cucumber (22.2%) and in roots of wheat (24.9%), maize (52.7%) and cucumber (32.2%) (Figure 7A). In contrast, inoculation did not significantly enhance free NH_4_^+^ concentration in plant tissues under high N (Figure 7B).

Similarly, *P. beijingensis* inoculation significantly increased the free NO_3_^-^ concentration in shoots of wheat (26.3%), maize (23.4%) and cucumber (43.7%) and in roots of wheat (60.7%), maize (62.6%) and cucumber (67.4%) under low N (Figure 7C). In contrast, inoculation did not significantly enhance free NO_3_^-^ concentration in plant tissues under high N (Figure 7D).

Compared to un-inoculated shoots under low N level, a significant increase of total N content was observed in inoculated shoots of wheat (76.3%), maize (49.1%) and cucumber (88.8%) (Figure 7E). Similarly, inoculation greatly enhanced total N content in inoculated roots of wheat (61.6%), maize (68.9%) and cucumber (92.3%) (Figure 7F). In contrast, the increased levels of total N content in root and shoots were lower under high N (Figure 7E,F). The data were consistent with the change of the concentrations of the free NH_4_^+^, and free NO_3_^-^ under low N and high N.

### Enhancement of GS and NR Activities of Plants by Inoculation With *P. beijingensis* 1-18

**FIGURE 8:**
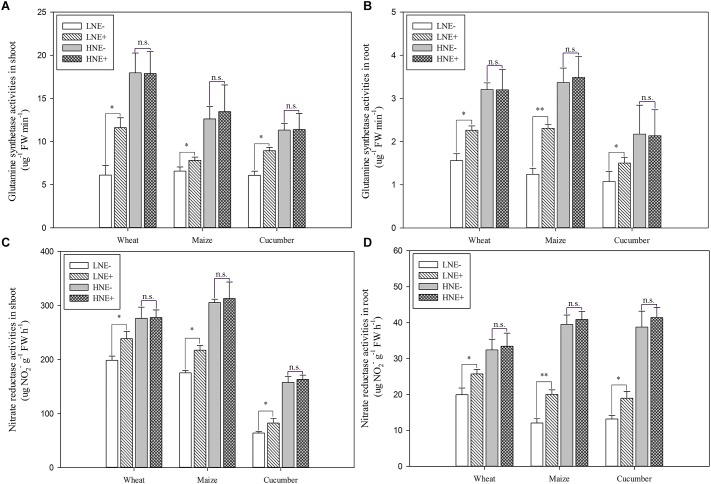
Glutamine synthetase (GS) (**A**: shoot; **B**: root) and Nitrate reductase (NR) (**C**: shoot; **D**: root) activities in shoots and roots of wheat, maize, and cucumber seedling inoculated with *P. beijingensis* BJ-18 under high N (HN) and low (LN) levels. Values are given as mean of three independent biological replicates, and single asterisks or double asterisks (^∗^ or ^∗∗^) indicate significant differences between inoculated (E+) and un-inoculated (E-) plants determined by LSD at *P* < 0.05 or *P* < 0.01. The bars represent the standard error. LNE- indicates plants grown in low N level of soil and un-inoculated with *P. beijingensis* BJ-18; LNE+ indicates plants grown in low N level of soils and inoculated with *P. beijingensis* BJ-18; HNE- indicates plants grown in high N level of soil and un-inoculated with *P. beijingensis* BJ-18; HNE+ indicates plants grown in high N level of soils and inoculated with *P. beijingensis* BJ-18.

Compared to un-inoculated plant shoots and roots under low N (Figure 8A,B), GS activities were significantly increased in inoculated shoots of wheat (89.7%), maize (19.0%) and cucumber (46.9%) and in inoculated roots of wheat (45.0%), maize (85.5%) and cucumber (40.4%), but inoculation did not obviously enhance the GS activities of plants grown in high N soil.

As shown in Figure 8C,D, compared to un-inoculated plant shoots and roots grown in low N soil, NR activities were significantly enhanced in inoculated shoots of wheat (20.9%), maize (42.0%) and cucumber (28.9%) and in inoculated roots of wheat (28.9%), maize (66.2%) and cucumber (44.3%). In contrast, inoculation did not obviously enhance the NR activities of plants grown in high N soil.

### Up-Regulation of Expression of N Uptake and N Assimilation Genes in Plants by Inoculation With *P. beijingensis*

**FIGURE 9:**
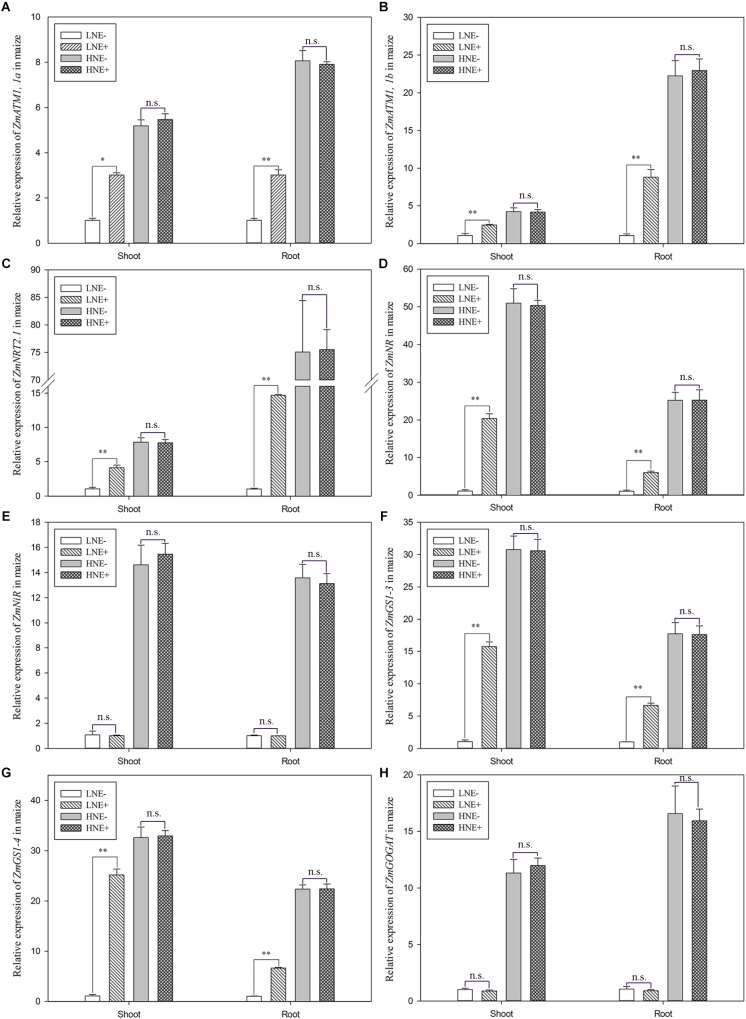
The transcript levels of genes involved N uptake (**A**: *ZmAMT1, 1a*; **B**: *ZmAMT1, 1b*; **C**: *Zm NRT2.1*) and assimilation (**D**: *ZmNR*; **E**: *ZmNiR*; **F**: *ZmGS1-3*; **G**: *ZmGS-4*; **H**: *ZmGOGAT*) in shoots and roots of maize seedlings inoculated with *P. beijingensis* BJ-18 under high N (HN) and low N (LN) levels. Values are given as mean of three independent biological replicates, and single asterisks or double asterisks (^∗^ or ^∗∗^) indicate significant differences between inoculated (E+) and un-inoculated (E-) plants determined by LSD at *P* < 0.05 or *P* < 0.01. The bars represent the standard error. LNE- indicates plants grown in low N level of soil and un-inoculated with *P. beijingensis* BJ-18; LNE+ indicates plants grown in low N level of soils and inoculated with *P. beijingensis* BJ-18; HNE- indicates plants grown in high N level of soil and un-inoculated with *P. beijingensis* BJ-18; HNE+ indicates plants grown in high N level of soils and inoculated with *P. beijingensis* BJ-18.

In maize, the transcript levels of three genes (*ZmAMT1*,*1a, ZmAMT1*,*1b*, and *ZmNRT2.1*) involved in N uptake and five genes (*ZmNR, ZmNiR, ZmGS1-3, ZmGS1-4*, and *Zm CsGOGAT*) involved N metabolism were analyzed (Figure 9). Under low N level, the transcript levels of *ZmAMT1*,*1a, ZmAMT1*,*1b*, and *ZmNRT2.1* were up-regulated by 1.83–3.93 folds in the inoculated shoots, while in inoculated roots, they were up-regulated 2.97–14.17 folds (Figure 9A–C), suggesting that inoculation promoted expression of plant N uptake genes. Similarly, inoculation significantly enhanced the transcript levels of *ZmNR, ZmGS1-3*, and *ZmGS1-4* in shoots by 18.46, 7.44, and 11.79 folds, respectively, and in roots by 5.63, 6.64, and 6.59 folds, respectively (Figure 9D,F,G). In contrast, inoculation did not obviously affect the transcript levels of these genes in maize shoots and roots under high N condition. However, inoculation did not affect the transcript levels of *ZmNiR* and *ZmGOGAT* under both low N and high N conditions (Figure 9E,H).

**FIGURE 10:**
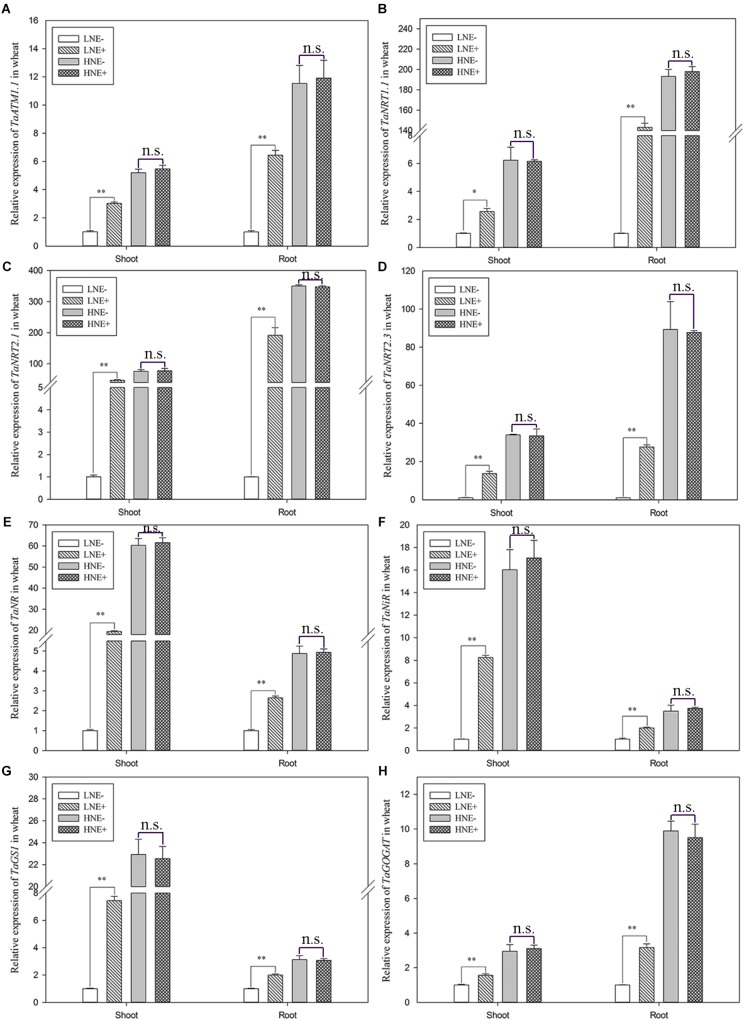
Transcript levels of genes involved N uptake (**A**: *TaAMT1.1*; **B**: *TaNRT1.1*; **C**: *TaNRT2.1*; **D**: *TaNRT2.3*) and assimilation (**E**: *TaNR*; **F**: *TaNiR*; **G**: *TaGS1*; **H**: *TaGOGAT*) in shoots and roots of wheat seedlings inoculated with *P. beijingensis* BJ-18 under high N (HN) and low N (LN) levels. Values are given as mean of three independent biological replicates, and single asterisks or double asterisks (^∗^ or ^∗∗^) indicate significant differences between inoculated (E+) and un-inoculated (E-) plants determined by LSD at *P* < 0.05 or *P* < 0.01. The bars represent the standard error. LNE- indicates plants grown in low N level of soil and un-inoculated with *P. beijingensis* BJ-18; LNE+ indicates plants grown in low N level of soils and inoculated with *P. beijingensis* BJ-18; HNE- indicates plants grown in high N level of soil and un-inoculated with *P. beijingensis* BJ-18; HNE+ indicates plants grown in high N level of soils and inoculated with *P. beijingensis* BJ-18.

In wheat, the transcript levels of four genes (*TaAMT1.1, TaNRT1.1, TaNRT2.1*, and *TaNRT2.3)* involved in N uptake and four genes (*TaNR, TaNiR, TaGS1*, and *TaGOGAT)* involved N metabolism were analyzed. Similar to maize, the eight genes in the inoculated shoots of wheat in low N were up-regulated by 1.56–46.49 folds, while they were up-regulated 1.98–91.93 folds in inoculated roots (Figure 10). In contrast, inoculation did not obviously affect the transcript levels of these genes under high N condition.

**FIGURE 11:**
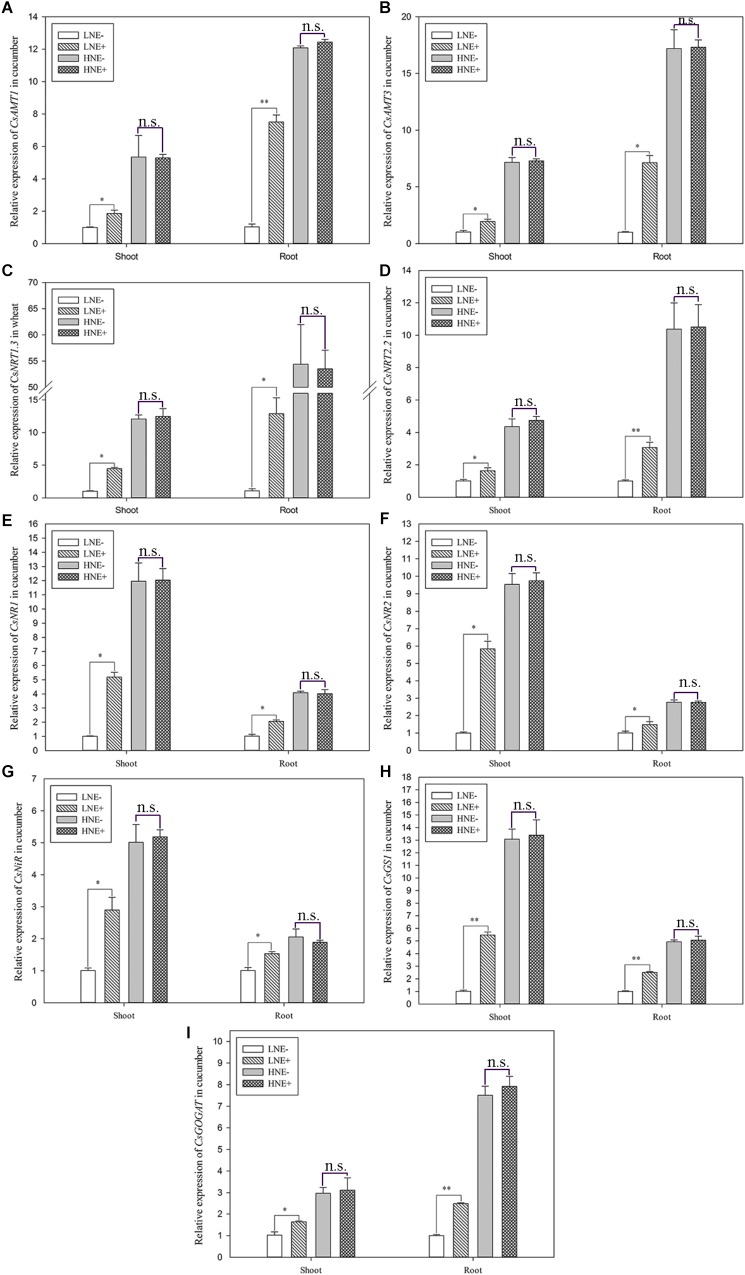
The transcript levels of genes involved N uptake (**A**: *CsAMT1*; **B**: *CsAMT3*; **C**: *CsNRT1.3*; **D**: *CsNRT2.2*) and assimilation (**E**: *CsNR1*; **F**: *CsNR2*; **G**: *CsNiR*; **H**: *CsGS1*; **I**: *CsGOGAT*) in shoots and roots of cucumber seedlings inoculated with *P. beijingensis* BJ-18 under high N (HN) and low N (LN) levels. Values are given as mean of three independent biological replicates, and single asterisks or double asterisks (^∗^ or ^∗∗^) indicate significant differences between inoculated (E+) and un-inoculated (E-) plants determined by LSD at *P* < 0.05 or *P* < 0.01. The bars represent the standard error. LNE- indicates plants grown in low N level of soil and un-inoculated with *P. beijingensis* BJ-18; LNE+ indicates plants grown in low N level of soils and inoculated with *P. beijingensis* BJ-18; HNE- indicates plants grown in high N level of soil and un-inoculated with *P. beijingensis* BJ-18; HNE+ indicates plants grown in high N level of soils and inoculated with *P. beijingensis* BJ-18.

In cucumber, the transcript levels of four genes (*CsAMT1, CsAMT3, CsNRT1.3*, and *CsNRT2.2*) involved in N uptake and five genes (*CsNR1, CsNR2, CsNiR, CsGS1*, and *CsGOGAT*) involved N metabolism were also analyzed. Similar to wheat and maize, the nine genes in the inoculated shoots of cucumber in low N soil were up-regulated 1.60–5.82 folds, while they were up-regulated 1.47–11.85 folds in inoculated roots (Figure 11). However, these effects of inoculated were weak when cucumber was grown in high N soil.

## Discussion

The N_2_-fixing *Paenibacillus* strains have gained much attention due to their capacity of forming endospore to survive for long periods of time under adverse conditions (Grady et al., 2016). In this study, *P. beijingensis* BJ-18 was tagged by GFP and observation by laser confocal microscopy revealed that in seedlings of wheat, maize, and cucumber, bacterial cells could be found in the inner cortex and vascular bundle of roots and stems as well as within the leaves, suggesting *P. beijingensis* BJ-18 has similar invasion patterns in both monocotyledons and dicotyledons. The data indicated that *P. beijingensis* BJ-18 spread systemically from roots to stems and leaves of wheat, maize, and cucumber via xylem vessels. Therefore, *P. beijingensis* BJ-18 could be defined as a plant endophytic diazotrophic bacterium and it had a broad range of host plants. Similar colonization patterns were observed in the association of *P. polymyxa* WLY78 with wheat, maize, and cucumber (Hao and Chen, 2017) and in the association of *P. polymyxa* P2b-2R with lodgepole pine (a gymnosperm tree species) (Anand and Chanway, 2013). The colonization pattern of *P. beijingensis* BJ-18 was a little different form that of the associated diazotrophic *A. brasilense* Yu62 which colonized mainly on the surface of maize roots and only small part entered into maize tissues (Liu et al., 2003). These results indicated that on one side the diazotrophic *Paenibacillus* species/strains may fix N_2_ inside plants and rapidly transfer the fixed product to plants, and on the other side they fix N_2_ on the root surfaces and part of the fixed product may be remain in the soil.

In this study, the concentrations of *P. beijingensis* cells in inoculated plant tissues under high N and low N levels were determined by qPCR, with un-inoculated plant tissues as control. The results showed that the bacterial cell numbers were significantly higher in inoculated plant tissues grown under low N condition than those under high N condition, suggesting that soil N status controlled the concentration of bacterial cells in plants. Similar reports were found in sugarcane where high dose of N (ammonium nitrate) resulted in reduction of endophytic *Acetobacter diazotrophicus* concentration (Fuentes-Ramirez et al., 1999) and a higher number of endophytic diazotrophs were isolated from sugarcane plants under low N than under high N (de Oliveira et al., 2003). Moreover, it was reported that the plant endogenous N status could also induce plant defense responses to regulate bacterial colonization (Carvalho et al., 2014). The increase in N compounds and amino acids (such as phenylalanine and hydroxyproline) was necessary to activate plant defense responses (Snoeijers et al., 2000). Amino acid transporters regulated by N status also have regulatory function in plant defense responses (Liu et al., 2010; Seifi et al., 2013). This study also showed that total N content and concentrations of NH_4_^+^ and NO_3_^-^ in un-inoculated plants under high N were much higher than those under low N. This finding may explain why high N caused a decrease in the numbers of *P. beijingensis* cells in plants tissues.

Furthermore, this study investigated whether *nifH* gene was expressed in the inoculated plants. The expression levels of *nifH* in the inoculated plant shoots and roots under low N were significantly higher than those under high N, consistent with the concentrations of *P. beijingensis* cells. It was well known that *nif* gene expression was inhibited by high concentrations of NH_4_^+^ (Dixon and Kahn, 2004). GlnR mediated positive and negative regulation of *nif* gene expression in *P. polymyxa* WLY78 according to N availably (Wang et al., 2018). As mentioned above, there were high concentrations of NH_4_^+^ in plants under high N. Thus, *P. beijingensis* cells in plant tissues sensed the N signal and then regulated *nifH* expression according to change of N levels. The expression of *nif* gene of *Herbaspirillum seropedicae* in maize, sorghum, wheat, and rice plants was reported (Roncato-Maccari et al., 2003). Similar report was found that N fertilizer application inhibited *nifH* gene expression of the endophytic diazotroph in sugarcane leaves (Jia Hui et al., 2017).

An important metric to evaluate the role of diazotrophic bacteria is whether they can provide fixed N_2_ to the host plant. The ^15^N isotope dilution analysis has been widely applied to quantify BNF in non-legume plant species such as rice inoculated with *Herbaspirillum seropedicae* Z67 (James et al., 2002), wheat with *Azospirillum brasilense* Wa5 (Christiansenweniger and Vanveen, 1991), Kallar grass with *Azoarcus* sp. BH72 (Hurek et al., 2002) and maize with *P. polymyxa* P2b-2R (Puri et al., 2015). Here, ^15^N isotope enrichment analysis method was used to estimate the contribution of BNF by *P. beijingensis* BJ-18 inoculation to plants. The BNF rates in the three different plants were higher under low N level than under high N level, indicating that BNF was affected by N levels. The data were consistent with the *nif* gene expression and the concentrations of *P. beijingensis* BJ-18 within the plant tissues under different N levels. Similar results were reported that sugarcane plants inoculated with diazotrophic strains gained higher N from BNF in the N-deficient soil (de Oliveira et al., 2003). This study also revealed that wheat gained the highest N from BNF under both low N and high N levels, followed by cucumber and then by maize, suggesting that BNF rate was related to host plant species. The shoots and roots of palm inoculated with *Bacillus sphaericus* UPMB-10 gained 13.2–13.4% N from BNF (Zakry et al., 2012), which was lower than that gained by inoculation with *P. beijingensis* BJ-18.

In this study, the effects of *P. beijingensis* BJ-18 inoculation on plant N uptake and metabolism were investigated. The concentrations of NO_3_^-^, NH_4_^+^ and total N were higher in roots and shoots of inoculated plants than in un-inoculated plants, and were higher in roots than in shoots. The positive effects were also controlled by soil N status. The results indicated that inoculation with *P. beijingensi*s BJ-18 promoted plants to uptake NO_3_^-^ and NH_4_^+^ from soil. The increased concentrations of NH_4_^+^ and total N in inoculated plants were at least partially resulted from BNF. Studies on the effects of diazotrophs on plant N uptake and metabolism were rare. However, inoculations with some endophytic fungi significantly improved N accumulation and metabolism were observed in rice (Yang et al., 2014), sugar beet (Shi et al., 2009), and tall fescue (Lyons et al., 1990).

NO_3_^-^ was mainly absorbed via NRT protein family members, and then transformed into NH_4_^+^ by NR. In this study, *P. beijingensis* BJ-18-inoculated plants showed significantly higher expression levels of *NRT* genes (*CsNRT1.3* and *CsNRT2.2* in cucumber; *TaNRT1.1, TaNRT2.1*, and *TaNRT2.3* in wheat; *ZmNRT2.1* in maize) in both shoots and roots under low N condition. Similar reports were found that under low N condition *NRT* genes were significantly up-regulated in rice inoculated with endophytic fungus *Phomopsis liquidambari* (Yang et al., 2014) and in tomato inoculated with diazotrophic *Enterobacter radicincitans* (Berger et al., 2013).

Higher NR activities were observed in the *P. beijingensis* BJ-18-inoculated plants. It was reported that inoculation with endophytic fungus *Plectosphaerella cucumerina* F11 greatly increased the activity of NR in sugar beet (Shi et al., 2009). To investigate whether the changes of NR activities in the *P. beijingensis* BJ-18- inoculated plants were closely related to differential expression of plant *NR* genes, the expression levels of *NR* genes were quantified. qRT-PCR analysis indicated that the expression levels of *NR* genes (*CsNR2* in cucumber, *TaNR* in wheat and *ZmNR* in maize) were significantly higher in inoculated plants than in un-inoculated plants under low N. It was reported that the endophytic fungus *Piriformospora indica* inoculation promoted N accumulation in *Arabidopsis* and tobacco seedlings by inducing the expression of *NR* (Sherameti et al., 2005). In contrast, endophytic fungus *Phomopsis liquidambari* inoculation significantly reduced NO_3_^-^ concentration in rice shoots under low N condition, since the higher NR activity made more NO_3_^-^ to be transformed into NH_4_^+^ (Yang et al., 2014).

This study demonstrated that *P. beijingensis* BJ-18-inoculated plants showed higher NH_4_^+^ concentration in roots and shoots, compared with those in un-inoculated plants. NH_4_^+^ was absorbed via AMT protein family members mainly, and then transformed into organic molecules by GS and GOGAT. GS is an important rate-limiting enzyme in NH_4_^+^ assimilation. The higher GS activities were observed in the *P. beijingensis* BJ-18-inoculated plants than those in un-inoculated plants. GS activities were closely related to soil N status. Transcript levels of *GS* genes were also measured to confirm whether the changes of GS activities in plants caused by *P. beijingensis* BJ-18 inoculation were related to *GS* genes transcription. qRT-PCR indicated that the higher expression levels of *GS* genes (*CsGS1, TaGS1, ZmGS1-3*, and *ZmGS1-4*) in inoculated plant tissues than in un-inoculated ones under low N. Similarly, it was also reported that the expression levels of *GS* genes were also significantly higher in inoculated plants with the endophytic fungus *P. liquidambari* than in un-inoculated plants under low N (Yang et al., 2014).

This study demonstrated that inoculation with *P. beijingensis* BJ-18 promoted dry weight of plant roots and shoots grown under low N to be increased by 20.3–103.6%. The current results were in agreement with previous reports that inoculation with *P. beijingensis* BJ-18 increased wheat yield by 26.9% in field experiment (Shi et al., 2016) and increased tomato shoot length, fresh weight, and dry weight in the pot experiments (Xie et al., 2016).

The plant-growth-promoting rhizobacteria facilitate plant growth by several direct and indirect mechanisms. Direct mechanisms include P solubilization, N fixation and hormone (e.g., IAA, cytokinins and gibberellins) production. Indirect mechanisms include controlling phytopathogens by producing antibiotics or lytic enzymes (Glick, 2012). As mentioned above, *P. beijingensis* BJ-18 provided N for plants by BNF and thus promoted plant growth. Also, this bacterium may promote plant growth by producing IAA and antimicrobial compounds (Xie et al., 2016). Compared to *A. brasilense* Yu62 which produced high amount of IAA, *P. beijingensis* BJ-18 produced a little amount of IAA. Thus, IAA produced by *P. beijingensis* BJ-18 might be not the major factor promoting plant growth. Since the soil used in greenhouse study was not sterile, the BNF/plant growth promotion observed in this study could have been in part due to one or several indigenous microbes present in that soil. This study for the first time revealed that *P. beijingensis* BJ-18 promoted plants to uptake N from soil and enhanced gene expression and enzyme activities involved in N uptake and assimilation in plants. In addition to BNF, these endogenous changes in plants induced by *P. beijingensis* BJ-18 might be another major factor promoting plant growth.

## Conclusion

This study demonstrated that *P. beijingensis* BJ-18 was an effective and endophytic diazotrophic bacterium which has similar colonization patterns in monocotylous and dicotyledonous plants. This bacterium promoted plant growth by direct mechanisms through BNF. Also, this bacterium might promote plant growth by indirect mechanisms through inducing endogenous changes in plants, including enhancement of N uptake and enzyme activities, and expression of N uptake and assimilation genes. The bacterial density within plant was closely related to the BNF efficiency and the endogenous changes in plants. However, the bacterial density, the BNF efficiency and the endogenous changes in plants during the association with *P. beijingensis* BJ-18 were controlled by the soil N status. These data suggested that successful colonization of *P. beijingensis* BJ-18 on plant was the first key step for this bacterium to promote plant growth by BNF and by inducing endogenous changes in plants. How soil N level affects bacterial colonization deserves further study.

## Author Contributions

YbL and SC designed and wrote the manuscript. YbL, YlL, HZ, and MW conducted the experiments.

## Conflict of Interest Statement

The authors declare that the research was conducted in the absence of any commercial or financial relationships that could be construed as a potential conflict of interest.

## References

[B1] Anand R. Chanway C. P. (2013). Detection of GFP-labeled *Paenibacillus polymyxa* in autofluorescing pine seedling tissues. *Biol. Fert. Soils* 49 111–118. 10.1007/s00374-012-0727-9

[B2] Baldani V. L. D. Baldani J. I. Dobereiner J. (2000). Inoculation of rice plants with the endophytic diazotrophs *Herbaspirillum seropedicae* and *Burkholderia* spp. *Biol. Fert. Soils* 30 485–491. 10.1007/s003740050027

[B3] Berger B. Brock A. K. Ruppel S. (2013). Nitrogen supply influences plant growth and transcriptional responses induced by *Enterobacter radicincitans* in *Solanum lycopersicum*. *Plant Soil* 370 641–652. 10.1007/s11104-013-1633-0

[B4] Bloom A. J. Sukrapanna S. S. Warner R. L. (1992). Root respiration associated with ammonium and nitrate absorption and assimilation by barley. *Plant Physiol.* 99 1294–1301. 10.1104/pp.99.4.1294 16669035 PMC1080623

[B5] Boddey R. M. Baldani V. L. Baldani J. I. Dobereiner J. (1986). Effect of inoculation of *Azospirillum* spp. on nitrogen accumulation by field-grown wheat. *Plant Soil* 95 109–121. 10.1007/bf02378857

[B6] Boddey R. M. Knowles R. (1987). Methods for quantification of nitrogen fixation associated with gramineae. *Crit. Rev. Plant Sci.* 6 209–266. 10.1080/07352688709382251

[B7] Boddey R. M. Oliveira O. C. D. Urquiaga S. Reis V. M. Olivares F. L. D. Baldani V. L. D. (1995). Biological nitrogen fixation associated with sugar cane and rice: contributions and prospects for improvement. *Plant Soil* 174 195–209. 10.1007/BF00032247

[B8] Carvalho T. L. Balsemao-Pires E. Saraiva R. M. Ferreira P. C. Hemerly A. S. (2014). Nitrogen signalling in plant interactions with associative and endophytic diazotrophic bacteria. *J. Exp. Bot.* 65 5631–5642. 10.1093/jxb/eru319 25114015

[B9] Christiansenweniger C. Vanveen J. A. (1991). NH4+-excreting *Azospirillum brasilense* mutants enhance the nitrogen supply of a wheat host. *Appl. Environ. Microb.* 57 3006–3012. 10.1128/aem.57.10.3006-3012.1991 PMC183912 16348569

[B10] de Oliveira A. L. M. Canuto E. D. Reis V. M. Baldani J. I. (2003). Response of micropropagated sugarcane varieties to inoculation with endophytic diazotrophic bacteria. *Braz. J. Microbiol.* 34 59–61. 10.1590/s1517-83822003000500020

[B11] Dixon R. Kahn D. (2004). Genetic regulation of biological nitrogen fixation. *Nat. Rev. Microbiol.* 2 621–631. 10.1038/nrmicro954 15263897

[B12] Eckhardt W. Bellmann K. Kolb H. (1999). Regulation of inducible nitric oxide synthase expression in β cells by environmental factors: heavy metals. *Biochem. J.* 338(Pt 3), 695-700. 10.1042/bj3400871u 10051441 PMC1220105

[B13] Fuentes-Ramirez L. E. Caballero-Mellado J. Sepulveda J. Martinez-Romero E. (1999). Colonization of sugarcane by *Acetobacter diazotrophicus* is inhibited by high N-fertilization. *FEMS Microbiol. Ecol.* 29 117–128. 10.1111/j.1574-6941.1999.tb00603.x

[B14] Galli V. Messias R. D. Silva S. Rombaldi C. V. (2013). Selection of reliable reference genes for quantitative real-time polymerase chain reaction studies in maize grains. *Plant Cell Rep.* 32 1869–1877. 10.1007/s00299-013-1499-x 24013792

[B15] Galloway J. N. Townsend A. R. Erisman J. W. Bekunda M. Cai Z. Freney J. R. (2008). Transformation of the nitrogen cycle: recent trends, questions, and potential solutions. *Science* 320 889–892. 10.1126/science.1136674 18487183

[B16] Glick B. R. (2012). Plant growth-promoting bacteria: mechanisms and applications. *Scientifica* 2012:963401. 10.6064/2012/963401 24278762 PMC3820493

[B17] Gordon S. A. Fleck A. Bell J. (1978). Optimal conditions for the estimation of ammonium by the berthelot reaction. *Ann. Clin.Biochem.* 15 270–275. 31129 10.1177/000456327801500164

[B18] Grady E. N. Macdonald J. Liu L. Richman A. Yuan Z. C. (2016). Current knowledge and perspectives of *Paenibacillus:* a review. *Microb. Cell Fact.* 15:203. 10.1186/s12934-016-0603-7 27905924 PMC5134293

[B19] Gupta V. Roper M. M. Roget D. K. (2006). Potential for non-symbiotic N2-fixation in different agroecological zones of southern Australia. *Aust. J. Soil Res.* 44 343–354. 10.1071/sr05122

[B20] Hao T. Chen S. (2017). Colonization of wheat, maize and cucumber by *Paenibacillus polymyxa* WLY78. *PLoS One* 12:e0169980. 10.1371/journal.pone.0169980 28076417 PMC5226731

[B21] Hurek T. Handley L. L. Reinhold-Hurek B. Piche Y. (2002). *Azoarcus* grass endophytes contribute fixed nitrogen to the plant in an unculturable state. *Mol. Plant Microbe Interact.* 15 233–242. 10.1094/mpmi.2002.15.3.233 11952126

[B22] James E. K. Gyaneshwar P. Mathan N. Barraquio Q. L. Reddy P. M. Iannetta P. P. M. (2002). Infection and colonization of rice seedlings by the plant growth-promoting bacterium *Herbaspirillum seropedicae* Z67. *Mol. Plant Microbe Interact.* 15 894–906. 10.1094/mpmi.2002.15.9.894 12236596

[B23] James E. K. Olivares F. L. De Oliveira A. L. M. Dos Reis F. B. Da Silva L. G. Reis V. M. (2001). Further observations on the interaction between sugar cane and *Gluconacetobacter diazotrophicus* under laboratory and greenhouse conditions. *J. Exp. Bot.* 52 747–760. 10.1093/jexbot/52.357.747 11413211

[B24] Jia Hui L. I. Yuan D. Jian Ming L. U. Yang L. T. Yang Rui L. I. Xing Y. X. (2017). Effects of nitrogen fertilizer on the expression of nifH gene of endophytic azotobacter in sugarcane leaves. *Biotechnol. Bull.* 33 100–106. 10.13560/j.cnki.biotech.bull.1985.20170002

[B25] Ke X. Feng S. Wang J. Lu W. Zhang W. Chen M. (2018). Effect of inoculation with nitrogen-fixing bacterium *Pseudomonas stutzeri* A1501 on maize plant growth and the microbiome indigenous to the rhizosphere. *Sys. Appl. Microbiol.* 42 248–260. 10.1016/j.syapm.2018.10.010 30477902

[B26] Kloepper J. W. Beauchamp C. J. (1992). A review of issues related to measuring colonization of plant-roots by bacteria. *Can. J. Microbiol.* 38 1219–1232. 10.1139/m92-202

[B27] Lea P. J. Miflin B. J. (2003). Glutamate synthase and the synthesis of glutamate in plants. *Plant Physiol. Bioch.* 41 555–564. 10.1016/s0981-9428(03)00060-3

[B28] Liu G. S. Ji Y. Y. Bhuiyan N. H. Pilot G. Selvaraj G. Zou J. T. (2010). Amino acid homeostasis modulates salicylic acid-associated redox status and defense responses in *Arabidopsis*. *Plant Cell* 22 3845–3863. 10.1105/tpc.110.079392 21097712 PMC3015111

[B29] Liu H. Carvalhais L. C. Crawford M. Singh E. Dennis P. G. Pieterse C. M. J. (2017). Inner Plant values: diversity, colonization and benefits from endophytic bacteria. *Front. Microbiol.* 8:2552. 10.3389/fmicb.2017.02552 29312235 PMC5742157

[B30] Liu Y. Chen S. F. Li J. L. (2003). Colonization pattern of *Azospirillum brasilense* Yu62 on maize roots. *Acta Bot. Sin.* 45 748–752. 16502291

[B31] Livak K. J. Schmittgen T. D. (2001). Analysis of relative gene expression data using real-time quantitative PCR and the 2(-Delta Delta C(T)) Method. *Methods* 25 402–408. 10.1006/meth.2001.1262 11846609

[B32] Lyons P. C. Evans J. J. Bacon C. W. (1990). Effects of the fungal endophyte *Acremonium coenophialum* on nitrogen accumulation and metabolism in tall fescue. *Plant Physiol.* 92 726–732. 10.1104/pp.92.3.726 16667341 PMC1062360

[B33] Magnani G. S. Didonet C. M. Cruz L. M. Picheth C. F. Pedrosa F. O. Souza E. M. (2010). Diversity of endophytic bacteria in Brazilian sugarcane. *Genet. Mol. Res.* 9 250–258. 10.4238/vol9-1gmr703 20198580

[B34] Mirza M. S. Ahmad W. Latif F. Haurat J. Bally R. Normand P. (2001). Isolation, partial characterization, and the effect of plant growth-promoting bacteria (PGPB) on micro-propagated sugarcane in vitro. *Plant Soil* 237 47–54.

[B35] Moloudi F. Navabpour S. Soltanloo H. Ramazanpour S. S. Sadeghipour H. (2013). Catalase and metallothionein genes expression analysis in wheat cultivars under drought stress condition. *J. Plant Mol. Breed.* 1 54–68.

[B36] Monteiro R. A. Balsanelli E. Wassem R. Marin A. M. Brusamarello-Santos L. C. C. Schmidt M. A. (2012). *Herbaspirillum*-plant interactions: microscopical, histological and molecular aspects. *Plant Soil* 356 175–196. 10.1007/s11104-012-1125-7

[B37] Murashige T. Skoog F. (1962). A revised medium for rapid growth and bio assays with tobacco tissue cultures. *Physiol. Plant.* 15 473–497.

[B38] Oliveira I. C. Brears T. Knight T. J. Clark A. Coruzzi G. M. (2002). Overexpression of cytosolic glutamine synthetase. Relation to nitrogen, light, and photorespiration. *Plant Physiol* 129 1170–1180. 10.1104/pp.020013 12114571 PMC166511

[B39] Puri A. Padda K. P. Chanway C. P. (2015). Can a diazotrophic endophyte originally isolated from lodgepole pine colonize an agricultural crop (corn) and promote its growth? *Soil Biol. Biochem.* 89 210–216. 10.1016/j.soilbio.2015.07.012

[B40] Rasmussen S. Parsons A. J. Bassett S. Christensen M. J. Hume D. E. Johnson L. J. (2007). High nitrogen supply and carbohydrate content reduce fungal endophyte and alkaloid concentration in *Lolium perenne*. *New Phytol.* 173 787–797. 10.1111/j.1469-8137.2006.01960.x 17286827

[B41] Reinhold-Hurek B. Hurek T. (1998). Life in grasses: diazotrophic endophytes. *Trends Microbiol.* 6 202–202. 10.1016/s0966-842x(98)01277-3 9587190

[B42] Reinhold-Hurek B. Hurek T. (2011). Living inside plants: bacterial endophytes. *Curr. Opin. Plant Biol.* 14 435–443. 10.1016/j.pbi.2011.04.004 21536480

[B43] Roncato-Maccari L. D. B. Ramos H. J. O. Pedrosa F. O. Alquini Y. Chubatsu L. S. Yates M. G. (2003). Endophytic *Herbaspirillum seropedicae* expresses nif genes in gramineous plants. *FEMS Microbiol. Ecol.* 45 39–47. 10.1016/s0168-6496(03)00108-9 19719605

[B44] Seifi H. S. Van Bockhaven J. Angenon G. Hofte M. (2013). Glutamate metabolism in plant disease and defense: friend or foe? *Mol. Plant Microbe Interact.* 26 475–485. 10.1094/mpmi-07-12-0176-cr 23342972

[B45] Sherameti I. Shahollari B. Venus Y. Altschmied L. Varma A. Oelmuller R. (2005). The endophytic fungus *Piriformospora indica* stimulates the expression of nitrate reductase and the starch-degrading enzyme glucan-water dikinase in tobacco and *Arabidopsis* roots through a homeodomain transcription factor that binds to a conserved motif in their promoters. *J. Biol. Chem.* 280 26241–26247. 10.1074/jbc.M500447200 15710607

[B46] Shi H. Li Y. Li P. Wang Z. Chen S. (2016). Effect of nitrogen-fixing *Paenibacillus* spp. on wheat yield. *J. China Agric. Univ.* 21 52–55.

[B47] Shi Y. Lou K. Li C. (2009). Effects of endophytic fungus on sugar content and key enzymes activity in nitrogen and sugar metabolism of sugar beet (*Beta vulgaris* L.). *Acta Agronomica Sinica* 35 946–951.

[B48] Snoeijers S. S. Perez-Garcia A. Joosten M. De Wit P. (2000). The effect of nitrogen on disease development and gene expression in bacterial and fungal plant pathogens. *Eur. J. Plant Pathol.* 106 493–506. 10.1023/a:1008720704105 27085087

[B49] Sugiura M. Georgescu M. N. Takahashi M. (2007). A nitrite transporter associated with nitrite uptake by higher plant chloroplasts. *Plant Cell Physiol.* 48 1022–1035. 10.1093/pcp/pcm073 17566055

[B50] Van Deynze A. Zamora P. Delaux P.-M. Heitmann C. Jayaraman D. Rajasekar S. (2018). Nitrogen fixation in a landrace of maize is supported by a mucilage-associated diazotrophic microbiota. *PLoS Biol.* 16:e2006352. 10.1371/journal.pbio.2006352 30086128 PMC6080747

[B51] Wang L. Y. Li J. Li Q. X. Chen S. F. (2013). *Paenibacillus beijingensis* sp nov., a nitrogen-fixing species isolated from wheat rhizosphere soil. *Antonie Van Leeuwenhoke* 104 675–683. 10.1007/s10482-013-9974-5 23912443

[B52] Wang T. S. Zhao X. Y. Shi H. W. Sun L. Li Y. B. Li Q. (2018). Positive and negative regulation of transferred nif genes mediated by indigenous GlnR in Gram-positive *Paenibacillus* polymyxa. *PLoS Genet.* 14:24. 10.1371/journal.pgen.1007629 30265664 PMC6191146

[B53] Wei L. Deng X.-G. Zhu T. Zheng T. Li P.-X. Wu J.-Q. (2015). Ethylene is involved in brassinosteroids induced alternative respiratory pathway in cucumber (*Cucumis sativus* L.) seedlings response to abiotic stress. *Front. Plant Sci.* 6:982. 10.3389/fpls.2015.00982 26617622 PMC4639706

[B54] Xie J. Shi H. Du Z. Wang T. Liu X. Chen S. (2016). Comparative genomic and functional analysis reveal conservation of plant growth promoting traits in *Paenibacillus polymyxa* and its closely related species. *Sci. Rep.* 6:21329. 10.1038/srep21329 26856413 PMC4746698

[B55] Yang B. Wang X. M. Ma H. Y. Jia Y. Li X. Dai C. C. (2014). Effects of the fungal endophyte *Phomopsis liquidambari* on nitrogen uptake and metabolism in rice. *Plant Growth Regul.* 73 165–179. 10.1007/s10725-013-9878-4 24972305

[B56] Yu X. Z. Zhang F. Z. (2012). Activities of nitrate reductase and glutamine synthetase in rice seedlings during cyanide metabolism. *J. Hazard. Mater.* 22 190–194. 10.1016/j.jhazmat.2012.05.027 22633925

[B57] Zakry F. A. A. Shamsuddin Z. H. Abdul Rahim K. Zawawi Zakaria Z. Abdul Rahim A. (2012). Inoculation of *Bacillus sphaericus* UPMB-10 to young oil palm and measurement of its uptake of fixed nitrogen using the 15N isotope dilution technique. *Microbes Environ.* 27 257–262. 10.1264/jsme2.ME11309 22446306 PMC4036051

[B58] Zhang W. Ding Y. Yao L. Liu K. Du B. (2013). Construction of gene knock-out system for *Paenibacillus* polymyxa SC2. *Wei Sheng Wu Xue Bao* 53 1258–1266. 24697098

